# Retzius‐sparing radical prosatectomy: First 200 Australian cases

**DOI:** 10.1002/bco2.489

**Published:** 2025-02-04

**Authors:** Troy Richard John Gianduzzo, Philip Ellard Dundee

**Affiliations:** ^1^ The Wesley Hospital Brisbane Australia; ^2^ The University of Queensland Brisbane Australia; ^3^ The Royal Melbourne Hospital Melbourne Australia; ^4^ The University of Melbourne Melbourne Australia

**Keywords:** functional recovery, prostate cancer, radical prostatectomy, Retzius‐sparing, robotics, urinary continence

## Abstract

**Objectives:**

The objective of this study is to report the first multi‐centred Australian series of 200 cases of Retzius‐sparing radical prostatectomy (RSRP).

**Patients and methods:**

Between April 2017 and June 2024, 200 RSRP procedures (197 robotic, three laparoscopic) were performed separately by the authors in five centres across two Australian cities (Melbourne, Victoria and Brisbane, Queensland). Data were collected prospectively with ethics committee approval (UCH‐HREC 2019.01.279) at weeks 1, 4–6, and three‐monthly. Exclusion criteria included prostate size >80 cc, significant middle lobe, large anterior tumour, previous TURP or any clinical factor deemed to make RSRP unsuitable. These criteria were relaxed as experience was gained.

**Results:**

Median (interquartile range) age, body mass index and PSA were 65 (60–70) years, 26 (25–29) kg/m^2^ and 5.2 (4.0–7.0) ng/mL. Low, intermediate and high D'Amico risk groups were 3.5%, 75.0% and 21.5%, respectively. Median (interquartile range) skin‐to‐skin operative time was 163 (125–210) min and blood loss 200 (100–350) mL. There were 17 (8.5%) Clavien–Dindo grade 1–2 complications and 8 (4%) grade 3 complications. Final pT stage was 60.5% pT2 and 39.5% pT3. The overall positive surgical margins (PSM) rate was 14.5% including 3.3% pT2 and 29.1% pT3. At 1 week post catheter removal 53.5% were pad‐free, increasing to 58.5% and 65.0% at 4 and 6 weeks, then 79.5%, 84.6%, 88.2% and 91.3% at 3, 6, 9 and 12 months, respectively. When a security pad is included, 71.5% and 85.5% of men were continent at 4 and 6 weeks, then 94%, 96%, 96% and 97% at 3, 6, 9 and 12 months, respectively. Three men required a suburethral sling and one an artificial urinary sphincter. Ninety of 140 (60.4%) preoperatively potent men were potent at 12 months with or without phosphodiesterase‐5 inhibitors.

**Conclusion:**

RSRP provides excellent early continence and can be introduced safely with good oncological results by experienced minimally‐ invasive surgeons.

## 1 INTRODUCTION

Incontinence post radical prostatectomy (RP) is a significant cause of patient morbidity post robotic‐assisted radical prostatectomy (RARP) with reported 12‐month incontinence rates ranging from 4% to 31%.^1^ Recent patient‐reported outcome measure (PROM) data of 2030 men undergoing RP from 26 UK centres found that at 12 months post RP only 65% of men were pad‐free.^2^ Post RP incontinence induces psychological distress and anxiety and worsens depression.^3^ These issues have led some federal government health authorities to advocate that patients seek therapies alternative to surgery.^4^ Additionally, there is increasing interest in focal therapy to maximise quality of life (QOL) outcomes.^5^ It therefore behoves surgeons to improve functional outcomes following surgery.

Retzius‐sparing radical prostatectomy (RSRP) aims to better preserve periprostatic anatomical structures and so improve continence outcomes.^6^ The initial series of 200 patients reported immediate continence in 92% of the first 100 patients and 90% of the second 100 patients.^7^ Several meta‐analyses have demonstrated improved early continence using the RSRP technique compared to the standard anterior approach with advantages possibly continuing for the long term.^8^, ^9^, ^10^, ^11^ However, concerns exist regarding higher rates of positive surgical margins (PSM).^10^

While RSRP is being adopted globally, with early results published across several continents, to date, no Australian data has been published.^12^ Herein we present data from the first published Australian multi‐centred series of RSRP.

## 2 METHODS

### 2.1 Technique

+Between April 2017 and June 2024, 200 (197 robotic and three laparoscopic) RSRP procedures were performed by two experienced robotic surgeons in five centres across two cities (PD, Melbourne, Victoria and TG, Brisbane, Queensland). The technique employed was based on the original description by Galfano et al.^6^ with minimal alteration. Briefly, initial access to the vasa and vesicles is achieved through a Montsourris rectovesical pouch peritoneal incision. Denonvilliers' fascia is subsequently incised, and the rectum reflected. Neurovascular bundle dissection (intrafascial, interfascial or extrafascial) is then commenced on each side. The right pedicle is then controlled with Weck Hem‐o‐lok clips (Teleflex, NC, USA) or metal clips and the right neurovascular bundle dissection completed, then the left pedicle is divided and the left bundle mobilised. The posterior and then anterior bladder neck are then divided, the apex mobilised, and the urethra divided. The vesicourethral anastomosis is then performed with a running braided or barbed monofilament suture.

### 2.2 Patients

Inclusion criteria were those patients with clinically localised prostate cancer deemed suitable for surgery. Initially, certain exclusion criteria were applied so that technically more favourable cases were selected. These criteria were then relaxed as confidence with the technique was gained. These criteria included prostate volume <80 cc, no significant middle lobe, no large anterior tumour, no previous transurethral resection of the prostate, body mass index (BMI) <35 kg/m^2^ or any factor where the operating surgeon deemed the patient to be unsuitable.

### 2.3 Data collection

All data were prospectively collected and reported with ethics committee approval (UCH‐HREC 2019.01.279). Preoperative data included date of date of surgery, age at surgery, BMI (kg/m^2^), erectile function defined by the ability to have penetrative intercourse with or without phosphodiesterase‐5 inhibitors, PSA (ng/mL), biopsy Gleason score and International Society of Urological Pathology (ISUP) group, D'Amico risk group and clinical T stage (cTNM 2017). Intraoperative data included skin‐to‐skin operative time, whether nerve‐sparing was performed and type of nerve‐sparing, and estimated blood loss (mL). Postoperative data included postoperative specimen Gleason score, pathological T stage (pTNM 2017), tumour volume, margin status as per the Prostate Cancer Outcomes Registry‐Australia and New Zealand criteria, tumour volume, potency defined as the ability to achieve penetrative intercourse with or without phosphodiesterase‐5 inhibitor assistance, and continence defined in pad usage per day. A security pad was defined as a pad that was used for psychological reassurance or for negligible leakage. Postoperative outcome data were collected at 1 week after the trial of void, 4–6 weeks post‐surgery, then 3–6 monthly depending on each surgeon's practice. Complications within 30 days were prospectively recorded according to the Clavien–Dindo classification.^13^

### 2.4 Statistical analysis

Continuous and categorical variables were reported using median and interquartile ranges, and frequencies and proportions, respectively. Kaplan–Meier methodology was used to plot continence and potency recovery. Summary statistics and Kaplan–Meier curves were calculated using Microsoft® Excel® 2016 MSO version 2405 (Microsoft Corporation, WA, USA) and JMP Statistical Discovery Software, version 18 (JMP Statistical Discovery LLC, NC, USA).

## 3 RESULTS

Table 1 details the demographic and operative data for the series. The series represents a typical RP population cohort with a median (IQR) age, BMI and PSA of 65 (10) years, 26 (4.25) kg/m^2^ and 5.2 (3) ng/mL, respectively. Only 4% of patients were ISUP 1, while the majority (82.5%) were ISUP 2 or 3, and 75% were D'Amico intermediate risk. Most men (77.5%) were potent and 97% of procedures involved nerve‐spring. The median (IQR) operative time and blood loss was 161 (85) min and 200 (250) mL.

**TABLE 1:** Pre‐ and intraoperative data.

Characteristic	Median or number	IQR or percentage
Age, years (median, IQR)	65	60–70
BMI, kg/m^2^ (median, IQR)	26	25–29
PSA, ng/mL (median, IQR)	5.2	4.0–7.0
ISUP grade group (*n*, %)		
ISUP 1	8	4
ISUP 2	112	56
ISUP 3	53	26.5
ISUP 4	10	5
ISUP 5	17	8.5
D'Amico risk (*n*, %)		
Low	7	3.5
Intermediate	150	75
High	43	21.5
Erectile dysfunction (*n*, %)		
Present	45	22.5
Absent	155	77.5
Operative time, minutes (median, IQR)	163	125–210
Estimated blood loss, mL (median, IQR)	200	100–350
Nerve‐sparing (*n*, %)		
Unilateral	33	16.5
Bilateral	161	80.5
Non‐nerve‐sparing	6	3

Complications are reported in Table 2. Clavien–Dindo grade 3 complications occurred in 4.0% of patients. There were no grade 4 or 5 complications. Specifically, there were no rectal injuries or ureteric injuries.

**TABLE 2:** Complications.

Clavien–Dindo grade	Number	Percentage
1–2		
Robot malfunction	1	0.5
Urinary retention	6	3.0
Urine leak	1	0.5
Wound infection	2	1.0
Haematuria/clot retention requiring irrigations	2	1.0
UTI/sepsis	2	1.0
Pulmonary oedema	1	0.5
DVT	1	0.5
Confusion	1	0.5
3		
Flexible cystoscopy for retention	4	2.0
Flexible cystoscopy for bladder spasms	1	0.5
Laparotomy for small bowel obstruction	1	0.5
Haemorrhage	1	0.5
Anastomotic stricture	1	0.5
Ureteric injury	0	0
Rectal injury	0	0
4	0	0
5	0	0

Table 3 reports the pathological data on the operative specimen. Upgrading was apparent whereby the percentage of patients with ISUP 1 disease decreased from 4% preoperatively to 0.5%, while the percentage of ISUP 4 and 5 disease increased from 13.5% to 16.5%. The majority of cases (83%) had intermediate risk disease. The overall PSM rate was 14.5%, comprising 3.3% for pT2 and 29.1% for pT3 disease.

**TABLE 3:** Pathological data.

Characteristic	Median or number	IQR or percentage
ISUP grade group (*n*, %)		
ISUP 1	1	0.5
ISUP 2	99	49.5
ISUP 3	67	33.5
ISUP 4	4	2
ISUP 5	29	14.5
Tumour volume, mL (median, IQR)	2.18	1.27–4.06
Positive surgical margins		
Overall	27/200	14.5
pT2	4/121	3.3
pT3	23/79	29.1

**FIGURE 1:**
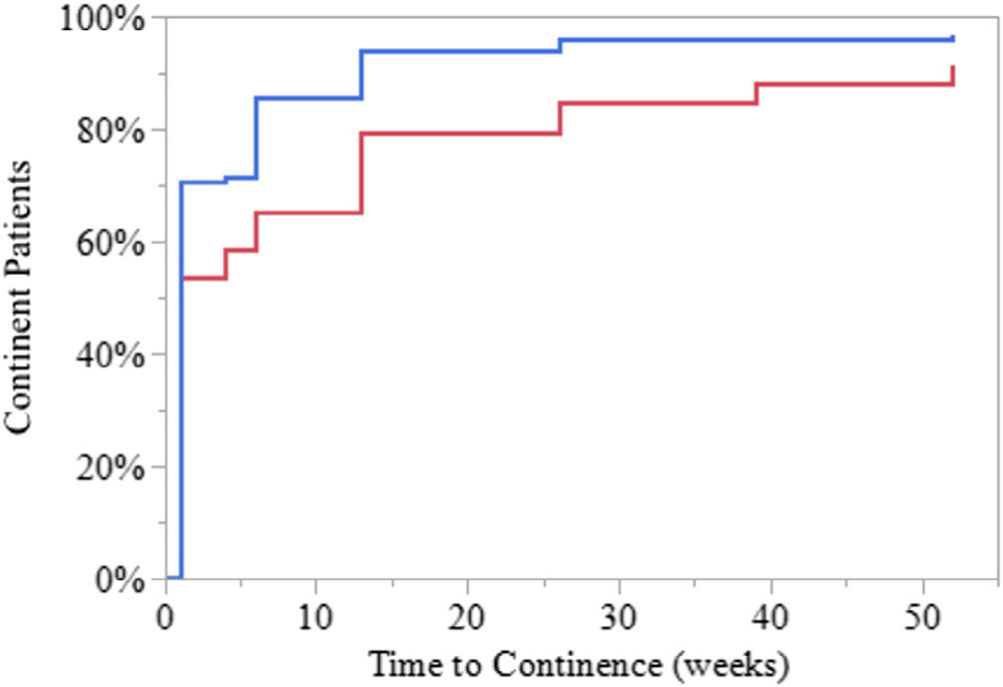
Kaplan–Meier curve of continence recovery. Red line is zero pads per day. Blue line includes the use of a ‘security’ pad.

Figure 1 plots a Kaplan–Meier curve of continence recovery. At 1 week post catheter removal 53.5% of men were pad‐free, increasing to 58.5% and 65.0% at 4 and 6 weeks, then 79.5%, 84.6%, 88.2% and 91.3% at 3, 6, 9 and 12 months, respectively. When the use of a ‘security’ pad is included, 71.5% and 85.5% of men were continent at 4 and 6 weeks, plateauing at 3 months at 94% then 96%, 96% and 97% at 6, 9 and 12 months, respectively. Three men were fitted with a suburethral sling, and one man required insertion of an artificial urinary sphincter. Ninety of 140 preoperatively potent men (60.4%) were potent at 12 months.

## 4 DISCUSSION

Incontinence remains a major issue post RP. The PROTECT study reported that at 12 months post RP, 36% of men were wearing pads, while the prostate cancer outcomes registry‐Victoria (PCOR‐Vic) reported 12‐month pad usage rates for open and robotic‐assisted surgery at 31.48% and 30.73%, respectively.^14^, ^15^ In the TrueNTH study, only 42% of men were leak‐free at 12 months with 9.7% of men reporting a ‘moderate or big problem’ with urine leak.^2^ Incontinence in the early postoperative period is particularly problematic. An extensive literature review of 193 618 open, laparoscopic and robotic RP patients revealed 1‐month pad‐free rates of 41%, 31% and 35%, respectively, and 61%, 64% and 69% at 3 months, increasing to 82%, 85% and 87% at 12 months.^16^ The majority of men in the TrueNTH study were incontinent in the first few months post RP.^2^ Similarly, the Australian randomised trial comparing open and robotic radical prostatectomy found that only 31% and 33% of men at 6 weeks and 66% and 60% of men at 12 weeks, respectively, were pad‐free.^17^

RSRP consistently demonstrates improved early continence results compared to the standard anterior technique.^9^, ^10^ Immediate pad‐free rates of 51%–76% are reported with RSRP, while at 3, 6 and 12 months 59%–92%, 82–97% and 90–100% of men are reported pad‐free.^11^ While concerns existing regarding higher PSM rates, the analysed series in the meta‐analyses are limited by small numbers and learning‐curve effects, and that the observed differences may not be apparent with increasing experience.^10^ In a propensity score matched analysis of 1863 patients, Lee et al. 2020 demonstrated no significant differences in PSM rates between RSRP and the standard approach as experience in RSRP is gained.^18^

The presented series is the first reported Australian series of RSRP. The demographic data are similar to previously published Australian series from the PCOR‐ANZ dataset with a preponderance of intermediate risk disease.^15^ The median operative time and blood loss of 163 min and 200 mL, respectively, are comparable to initial published series.^9^, ^10^, ^11^ Major complications were rare as only 4.0% of the cohort had Clavien–Dindo grade 3 or higher complications. Significantly, there were no ureteric or rectal injuries.

Early continence was excellent with a pad‐free rate of 53.5% within the first week of catheter removal. This pad‐free rate rapidly plateaued so that by 3 months 79.5% of men were pad‐free. Of the remaining 20.5% of men not pad‐free by 3 months, incontinence was minimal, whereby 14.5% were wearing a ‘security’ pad, and only 6% of men were wearing two or more pads per day. Four men required corrective surgery for long‐term refractory incontinence. This series did not examine comparative data against the standard anterior approach. However, these continence outcomes are consistent with the Cochrane review conclusion that by 12 months, there may be little difference in continence rates between RSRP and the standard technique.^8^ The potency rate of 60.4% is comparable to previously published series.^19^ The overall PSM rate of 14.5% with a pT2 rate of 3.3% and a pT3 rate of 29.1% compares favourably to the early published RSRP data and to the 15% overall PSM rate in the robotic arm of the Australian open and robotic radical prostatectomy randomised trial.^11^, ^17^

The strength of this study is that it is the first report of RSRP in the Australian healthcare system. In addition, it is a multi‐centred study undertaken by two surgeons in two states. The main limitation of the series is that is that it does not directly compare RSRP outcomes against the standard anterior approach. However, there have been several such publications, and the presented results are comparable to those reports. Additionally, learning‐curve effects have not been specifically examined and are the subject of another paper. Opportunity exists for further multi‐centred Australian collaboration to gain further insights into RSRP in the Australian setting.

In conclusion, this is the first reported Australian series of RSRP. It demonstrates that RSRP can be safely introduced into the Australian healthcare system by surgeons experienced in RARP. Excellent early continence consistent with published international series was achieved with good oncological control. However, further data are required to determine whether these early continence results translate into a sustained long‐term advantage.

## AUTHOR CONTRIBUTIONS

Both T. R. J. Gianduzzo and P. E. Dundee have substantially contributed to the authorship of this paper. Both authors performed the surgical procedures and contributed to the conception and design of the work and to the acquisition and interpretation of the data. T. R. J. Gianduzzo performed the data analysis and drafted the work. Both authors critically reviewed the work for intellectual content, and both gave final approval of the version to be published. Both authors agree to be accountable for all aspects of the work.

## CONFLICT OF INTEREST STATEMENT

The authors declare there are no conflicts of interest.
